# Combined PI3K Inhibitor and Eribulin Enhances Anti-Tumor Activity in Preclinical Models of Paclitaxel-Resistant, PIK3CA-Mutated Endometrial Cancer

**DOI:** 10.3390/cancers15194887

**Published:** 2023-10-08

**Authors:** Yeong Gyu Jeong, Nar Bahadur Katuwal, Min Sil Kang, Mithun Ghosh, Sa Deok Hong, Seong Min Park, Seul-Gi Kim, Tae Hoen Kim, Yong Wha Moon

**Affiliations:** 1 Department of Biomedical Science, The Graduate School, CHA University, Seongnam-si 13488, Republic of Korearkdalstlf1097@gmail.com (M.S.K.);; 2 Hematology and Oncology, Department of Internal Medicine, CHA Bundang Medical Center, CHA University, Seongnam-si 13496, Republic of Korea; sophia311@chamc.co.kr; 3 Department of Pathology, CHA Bundang Medical Center, CHA University, Seongnam-si 13496, Republic of Korea

**Keywords:** chemotherapy, PI3K pathway, endometrial cancer, drug resistance, paclitaxel resistance, eribulin, alpelisib

## Abstract

**Simple Summary:**

Paclitaxel-based chemotherapy is the standard front-line therapy for advanced or metastatic endometrial cancer. However, paclitaxel resistance eternally develops. Based on the high prevalence of PIK3CA mutation, reaching 50%, in endometrial cancer, we preclinically investigated the effectiveness of a combination of a PI3K inhibitor with eribulin, a post-paclitaxel therapy for breast cancer, in treating paclitaxel-resistant, PIK3CA-mutated endometrial cancer. Based on our in vitro and in vivo results, we suggest that paclitaxel resistance is associated with the activation of the PIK3/AKT pathway in PIK3CA-mutated endometrial cancer, and the combination of a PI3K inhibitor and eribulin merits further clinical investigation.

**Abstract:**

Endometrial cancer stands as the predominant gynecological malignancy in developed nations. For advanced or recurrent disease, paclitaxel-based chemotherapy is the standard front-line therapy. However, paclitaxel resistance eternally develops. Based on the high prevalence of phosphatidylinositol-4,5-bisphosphate 3-kinase catalytic subunit alpha (PIK3CA) mutation, reaching 50%, in endometrial cancer, we preclinically investigated the effectiveness of a combination of a phosphatidylinositol 3-kinase (PI3K) inhibitor with eribulin, a post-paclitaxel therapy for breast cancer, in treating paclitaxel-resistant, PIK3CA-mutated endometrial cancer. We generated paclitaxel-resistant cell lines from PIK3CA-mutated endometrial cancer cell lines by gradually increasing the concentration of paclitaxel in cell cultures. We observed that the PI3K/AKT and epithelial–mesenchymal transition (EMT) pathways in paclitaxel-resistant cells were significantly upregulated compared with those in parental cells. Then, we demonstrated that the combination of alpelisib (a PI3K inhibitor) and eribulin more effectively suppressed the cellular growth of paclitaxel-resistant cells in in vitro and in vivo xenograft models. Mechanistically, we demonstrated that the effect of the combination could be enhanced by inhibiting both the PI3K/AKT and EMT pathways. Therefore, we suggest that paclitaxel resistance is associated with the activation of the PIK3/AKT pathway in PIK3CA-mutated endometrial cancer, and the combination of a PI3K inhibitor and eribulin merits further clinical investigation.

## 1. Introduction

Endometrial cancer is the most common gynecological malignancy in developed countries and one of the major causes of cancer mortality among women. Endometrial cancer incidence is increasing due to an increase in risk factors, particularly obesity and aging [1,2]. In 1983, Bokhman categorized endometrial cancer into type I and type II based on clinical and hormonal features [2]. However, in 2020, the World Health Organization provided a histological classification for endometrial carcinoma, which includes endometrioid, serous, clear cell, mixed cell adenocarcinoma, and other relatively rare types [3,4].

The prognosis for recurrent endometrial cancer is poor. As a standard of care, advanced or recurrent disease is treated with paclitaxel/platinum chemotherapy. Mismatch repair (MMR) status should be assessed for optimal subsequent-line therapy after progression on first-line chemotherapy in this population. For those with microsatellite instability-high (MSI-H) or MMR-deficient (MMRd) endometrial cancer who have progressed on paclitaxel/platinum chemotherapy, an immune-checkpoint inhibitor such as pembrolizumab or dostarlimab is an appropriate option; however, if the tumors are not MSI-H or MMRd, the combination of pembrolizumab and the vascular endothelial growth factor receptor inhibitor lenvatinib has been approved by the Food and Drug Administration [1]. However, except for paclitaxel/platinum chemotherapy and immune checkpoint inhibitors with or without lenvatinib, treatment options for patients with advanced endometrial carcinoma are limited. Therefore, we need to explore therapeutic targets and develop more targeted therapies in endometrial cancer.

The phosphatidylinositol 3-kinase (PI3K)/protein kinase B (AKT) pathway has been reported to be activated and associated with chemotherapy resistance in several types of human cancer [5]. Several studies have reported that approximately 25–53% of endometrial cancer cases had PIK3CA mutations [6]. In more detail, depending on the type of endometrial cancer, particularly in type I endometrial cancer, the most frequent mutations associated with the PI3K pathway are phosphatase and tensin homolog (PTEN) loss (77%) and PIK3CA mutation (53%) [7]. In contrast, in type II serous endometrial cancer, PIK3CA mutation was found in 23.7% of cases [8]. Despite frequent alterations in the PI3K pathway in endometrial cancer, no PI3K inhibitors have been approved to date for endometrial cancer. This inspired us to study PI3K inhibitors in endometrial cancer.

In addition to this, numerous previous studies demonstrated that epithelial–mesenchymal transitions (EMTs) are associated with chemoresistance in several type of cancers, for example, pancreatic cancer, non-small cell lung cancer, colorectal cancer, breast cancer, and ovarian cancer [9]. However, EMT-associated mechanisms of resistance are not fully uncovered. EMT is a biological process in which cells switch from an epithelial state to a mesenchymal state. This involves decreasing the expression of epithelial markers like E-cadherin and increasing the expression of mesenchymal markers such as N-cadherin, vimentin, Snail, LAMC2, and ZEB1. EMT also activates several signaling pathways, such as TGFβ, NF-kB, Wnt, and Hedgehog. Blocking of the EMT pathway is critical for preventing cell migration and invasion as well as for restoring drug sensitivity [9,10]. However, the role of EMT in endometrial cancer is poorly studied [11].

To search for potential alternate chemotherapies for paclitaxel-resistant endometrial cancer, we turned our eyes to other cancers in a similar situation. Eribulin has been approved in soft tissue sarcoma [12] and metastatic breast cancer [13]; however, it has rarely been studied in endometrial cancer. Because eribulin is effective in patients with breast cancer who fail to respond to paclitaxel, the effectiveness of eribulin in treating other types of cancers progressing on paclitaxel may be worth studying. After paclitaxel/platinum is used as the first-line therapy in advanced or metastatic endometrial cancer, eribulin may be evaluated in such a setting.

Based on there being neither therapeutic options for paclitaxel-resistant endometrial cancer nor PI3K inhibitors approved in endometrial cancer, we established a preclinical model with paclitaxel-resistant, PIK3CA-mutated endometrial cancer. Using our preclinical model, we investigated the effectiveness of the combination of a PI3K inhibitor with eribulin, a post-paclitaxel therapy for breast cancer, demonstrating the synergistic anticancer activity of the combination. In this study, we provide a scientific rationale to further clinically develop this combination in endometrial cancer.

## 2. Materials and Methods

### 2.1. Drugs

Paclitaxel (catalog no. CS-1145) and alpelisib (catalog no. CS-0663) were purchased from ChemScene (Monmouth Junction, NJ 08852, USA). Eribulin was provided by Eisai Co., Ltd. (Bunkyo City, Tokyo, Japan). Paclitaxel and alpelisib were reconstituted in dimethyl sulfoxide (DMSO) (Sigma-Aldrich, Darmstadt, Germany).

### 2.2. Cell Culture

The endometrial cancer cell lines HEC1A (type I, PIK3CA G1049R mutated), HEC1B (type I, PIK3CA G1049R mutated), and AN3CA (type II, PTEN loss) were obtained from the American Type Culture Collection (Manassas, VA, USA). HEC1A and HEC1A-TR cells were maintained in Dulbecco’s Modified Eagle Medium (catalog no. LM001-05; Welgene Inc., Deagu, Korea), whereas AN3CA, HEC1B, and HEC1B-TR were maintained in Minimum Essential Medium (catalog no. LM007-07; Welgene Inc., Deagu, Korea). Briefly, 10% heat-inactivated FBS (catalog no. S001-01; Welgene Inc., Deagu, Korea) and 1% 100 × P/S solution (catalog no. LS202-02; Welgene Inc.) was added to all basal media.

### 2.3. Generation of Paclitaxel-Resistant Endometrial Cancer Cells

Paclitaxel-resistant cells were generated through a gradual increment in paclitaxel concentration, commencing at 1/100th of the maximal inhibitory concentration (IC_50_). Paclitaxel IC_50_ of HEC1A and HEC1B cell line was 16 nM and 15 nM, respectively. Cells were consistently cultured in media containing paclitaxel, and drug concentration was increased when cells could proliferate without restraint in the presence of the drugs. After 9–11 months, IC_50_ concentration was increased by >10-fold in both cells and named HEC1A-TR and HEC1B-TR, indicating paclitaxel resistance. In order to use resistant cells for assays, HEC1A-TR and HEC1B-TR cells were maintained with medium containing 5 nM and 15 nM paclitaxel, respectively. Prior to 48–72 h of conducting experiments, the medium containing the drug was replaced with drug-free medium.

### 2.4. Cell Viability Assay

Cell viability was measured using a thiazolyl blue tetrazolium bromide (MTT) (Sigma, St. Louis, MO, USA) colorimetric assay. In total, 1500–3000 cells/well in 100 µL medium with 10% FBS were added to the wells of a 96-well plate and incubated overnight. The cells were treated with different concentrations of alpelisib alone, eribulin alone, or their combination for 72 h. After drug treatment, the MTT solution was added to each well, and the plates were incubated for 4 h at 37 °C; then, the medium was removed. After dissolving the formazan crystals that were formed in 100 µL of DMSO (catalog no. DR1022-500-00; Biosesang., Yongin, Korea), the absorbance of each plate was measured at 540 nm using a Multiskan GO Microplate Spectrophotometer (Thermo-Fisher Scientific, Vantaa, Finland). IC_50_ and combination index (CI) values of alpelisib or eribulin were analyzed using CompuSyn (ComboSyn, Inc., Paramus, NJ, USA).

### 2.5. Western Blot

Protein samples were prepared by lysing cells in RIPA buffer (catalog no. 89901, Thermo-Fisher Scientific) containing phosphatase inhibitors (catalog no. 1862495, Thermo-Fisher Scientific) and protease inhibitor cocktail (catalog no. 11873580001, Roche) and were stored at 4 °C for 30 min. After centrifugation (14,000 rpm) at 4 °C for 15 min, the supernatant was collected. Protein concentrations were analyzed using a PierceTM BCA protein assay kit (catalog no. 23227, and Cat. No. 2322, Thermo-Fisher Scientific). Proteins in whole-cell lysates (20 µg) were prepared with SDS-PAGE loading buffer (catalog no. CBS002, LPS solution) and denatured at 100 °C for 5 min. They were separated using sodium dodecyl sulfate-polyacrylamide gel electrophoresis and electrotransferred onto polyvinylidene difluoride (catalog no. IPVH00010, MERCK) membranes. The membranes were blocked for 1 h in Tris-buffered saline containing 5% milk and 0.1% Tween 20 at room temperature. The primary antibody was added following washing with 1× Tris-Buffered Saline and 0.1% Tween^®^ 20 Detergent (TBST) and was incubated overnight at 4 °C with gentle shaking. A list of antibodies is shown in Supplementary Table S1.

On the following day, membranes were rinsed 10 min with TBST 3 times and then subjected to incubation with a secondary antibody conjugated with horseradish peroxidase for 1 h at ambient temperature.

Detection was performed with a Detection Reagent 1 peroxide solution (catalog no. 1859700, Thermo-Fisher Scientific) and Detection Reagent 2 Luminol Enhancer Solution (catalog no. 1859697, Thermo-Fisher Scientific) and imaged using a detection instrument with GeneSys.

### 2.6. Quantitative Real-Time Polymerase Chain Reaction (qRT-PCR)

Total RNA was extracted from the cells using TRIzol (Life Technologies). Total RNA was reverse transcribed to cDNA using the Takara prime script 1st strand cDNA synthesis kit (catalog no. 6110A, Takara Bio Inc.), according to the manufacturer’s instructions. qRT-PCR was performed using an ABI StepOne Real-Time PCR System (Applied Biosystems, Warrington, UK) and a Power-up SYBR Green Master Mix (catalog no. A25741, Thermo-Fisher Scientific). The expression of the target gene was normalized to that of glyceraldehyde 3-phosphate dehydrogenase (GAPDH). Primer sequences for GAPDH were as follows: 5′-CAGCCTCAAGATCATCAGCA-3′ (forward) and 5′-TGTGGTCATGAGTCCTTCCA-3′ (reverse). PIK3CA primers were as follows: 5′-AACAATGCCTCCACGACCAT-3′ (forward) and 5′-TCACGGTTGCCTACTGGTTC-3′ (reverse).

### 2.7. Migration and Invasion Assay

To perform the migration and invasion assay, SPLInsertTM Hanging plate (catalog no. 37224) with a pore size of 8.0 μm was used. For migration assay, 800 μL of complete media containing 10–15% FBS was added to the lower well and 200 μL FBS-free medium containing 4 × 10^5^ cells was seeded in the upper insert wells and incubated for 24 h.

Similarly, for invasion assay, we coated the insert well with 50 μL of Matrigel matrix (catalog no. 254234) and let it dry overnight at 37 °C incubation. Following incubation, 800 μL of complete medium containing 10–15% FBS was added to the lower well and 200 μL FBS-free medium containing 4 × 10^5^ cells was seeded in the upper insert wells and incubated for 24 h.

To analyze the impact of drugs on migration and invasion assay, IC_50_ concentration of each drug was treated for 24 h in lower well. After completion of the designated incubation times for migration and invasion assays, medium was carefully removed from both lower and insert wells. Furthermore, matrigel was removed from the invasion assay insert well along with the unmigrated cells. Similarly, only unmigrated cells were removed from the migration assay insert well using a cotton swab. The migrated and invasive cells were fixed using 2% PFA, followed by crystal violet staining for 15 min. Subsequently, insert wells were washed with PBS and water and allowed to dry for at least 3–4 h. Migrated and invaded cells were observed under the light microscope and five non-overlapping microscopic fields images were taken at 100× magnification. ImageJ software version 1.53t was used to quantify the migratory and invasive cells.

### 2.8. Apoptosis Assay

To assess the apoptosis rate following drug treatment, staining was carried out using APC annexin V (catalog no. 640920, BioLegend) and propidium iodide (PI) (catalog no. P3566, Sigma-Aldrich). After 72 h of drug treatment, cells were collected, washed with phosphate-buffered saline (PBS), and 400 μL annexin V 1× binding buffer (catalog no. 422201, BioLegend) was added. Furthermore, APC annexin V and PI (50 μg/mL) were added to the suspended cells and incubated for 15 min at ambient temperature in the dark. After 15 min, apoptosis cells were analyzed using flow cytometry with a total of 10,000 events recorded using a Beckman Coulter CytoFLEX instrument.

### 2.9. Animal Study

Mice were housed in a specific pathogen-free animal facility at CHA University (Seongnam, Korea) with a temperature of 24 ± 3 °C, a 12 h light/dark cycle, and with unlimited access to food and water. All mice were allowed to acclimate for at least a week before the experiment. All animal procedures were conducted by following the CHA University IACUC protocols. To establish tumor, 1 × 10^7^ HEC1B-TR cells in a mixture of Matrigel solution (corning, NY, USA) were injected subcutaneously at right flank. Once tumor volume reached 70–80 mm^3^, mice were randomly divided into four groups, each containing five mice. Each group of mice were treated with PBS, alpelisib, eribulin, or combination of both drugs. Tumor size was measured every two days using a caliper, and volume was calculated using a formula (tumor length × tumor width^2^ × 0.5). Body weight was also monitored every two days. After 24 days, mice were euthanized, and the xenografted tumors were collected and preserved for further analysis.

### 2.10. Statistical Analysis

All statistical analyses were performed using SPSS, version 19.0 (IBM Corp., Armonk, NY, USA). Student’s *t*-test was used to compare the two groups in qRT-PCR, apoptosis assay, western blot, and animal study. All *p*-values were two-sided, and *p*-values ≤ 0.05 were considered statistically significant.

## 3. Results

### 3.1. Activation of the PI3K/AKT and EMT Pathways Is Associated with Paclitaxel Resistance in Endometrial Cancer Cells

**Figure 1:**
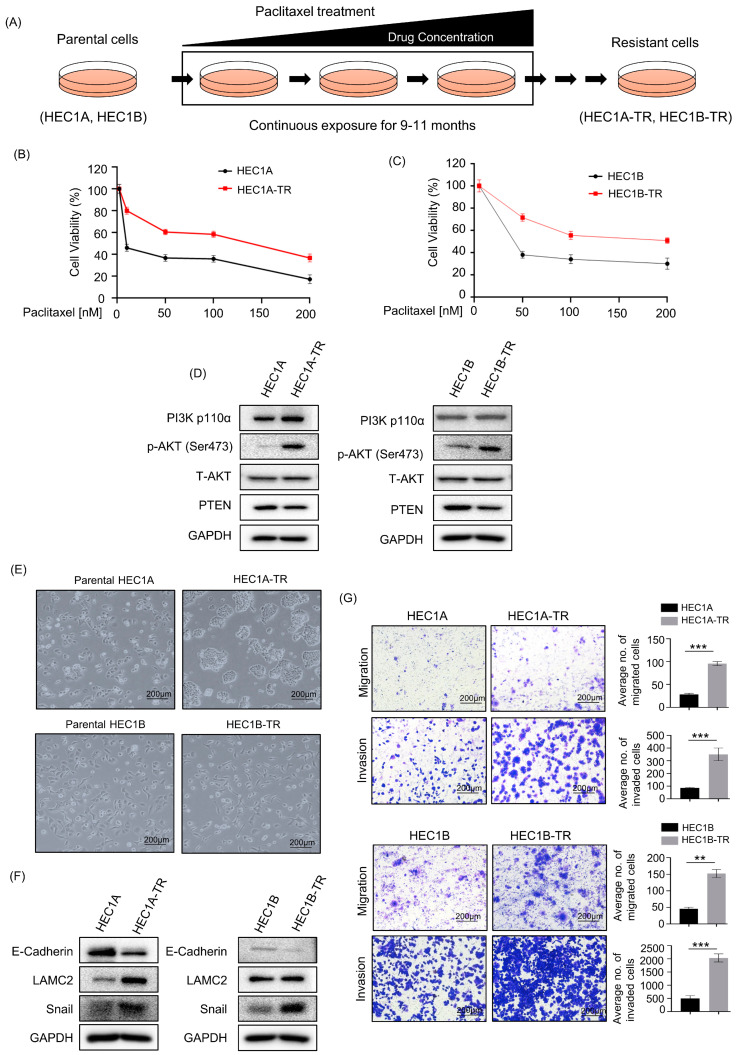
Activation of PI3K pathway and EMT pathway is associated with paclitaxel resistance in endometrial cancer cells. (**A**) Experimental design used to generate paclitaxel-resistant endometrial cancer cell lines (HEC1A-TR, HEC1B-TR). (**B**,**C**) The proliferation assay with paclitaxel in parental (HEC1A, HEC1B) and paclitaxel-resistant cells (HEC1A-TR, HEC1B-TR). Each parental and resistant cell line was treated with paclitaxel for 72 h. IC_50_ values were calculated using CompuSyn. Three independently repeated experiments were performed with similar results. (**D**) Western blots showing changes in expression of PI3K pathway-related genes in parental (HEC1A, HEC1B) and paclitaxel-resistant cells (HEC1A-TR, HEC1B-TR). Uncropped western blots are included in Figure S4. (**E**) Morphology of parental (HEC1A, HEC1B) and paclitaxel-resistant endometrial cancer cells (HEC1A-TR, HEC1B-TR). Parental cells had normally cobblestone-like epithelial morphology, and paclitaxel-resistant cells had mesenchymal morphology of spindle and fiber shapes. Original magnification was ×100. (**F**) Western blots showing protein expression of mesenchymal markers (E-cadherin, ZEB1, LAMC2, and Snail), and epithelial marker (E-cadherin) in HEC1A-TR and HEC1B-TR cells compared with parental HEC1A and HEC1B cells. (**G**) Migration and invasion assay of parental (HEC1A and HEC1B) versus paclitaxel-resistant cells (HEC1A-TR and HEC1B-TR). The number of migratory and invading cells from three different non-overlapping 100 × microscopic fields is expressed as mean ± SD in the right panel. Independent sample *t*-test: ** *p* < 0.01, *** *p* < 0.001.

Paclitaxel-resistant cells were generated from the PIK3CA-mutated, type I endometrial cancer cell lines HEC1A and HEC1B. As mentioned, paclitaxel IC_50_ concentration of paclitaxel-resistant cells (i.e., HEC1A-TR and HEC1B-TR) was significantly high compared with their corresponding parental cells; a 16.3-fold increase in HEC1A-TR and a 10.7-fold increase in HEC1B-TR compared with their counterpart parental cells (Figure 1B,C and Supplementary Table S2). Previous studies have shown that activation of the PI3K/AKT pathway is associated with drug resistance [14]. Furthermore, western blot analysis showed the upregulation of p-AKT without significant alteration in PI3K p110α and downregulation of PTEN in paclitaxel-resistant cells compared with those in parental cells (Figure 1D), indicating activation of the PI3K/AKT pathway in paclitaxel-resistant cells. Regarding the morphology of the cells, paclitaxel-resistant cells looked more like mesenchymal types than their corresponding parental cells, which looked like epithelial types (Figure 1E). Western blot further confirmed an increase in mesenchymal markers, such as Snail and LAMC2, but a decrease in E-cadherin, an epithelial marker, in paclitaxel-resistant cells, indicating EMT (Figure 1F). Moreover, EMT characteristics were confirmed with migration and invasion assays, where both paclitaxel-resistant cells (HEC1A-TR and HEC1B-TR) showed significant increases in migratory and invasive capacity compared with their corresponding parental cells (HEC1A and HEC1B) (Figure 1G). Taken together, these results suggest that paclitaxel resistance in PIK3CA-mutated endometrial cancer cells is associated with the activation of the PI3K/AKT and EMT pathways.

### 3.2. Alpelisib, a PI3K Inhibitor, in Combination with Eribulin Synergistically Inhibits the Proliferation of Paclitaxel-Sensitive and Paclitaxel-Resistant, PIK3CA-Mutated Endometrial Cancer Cells

**Figure 2:**
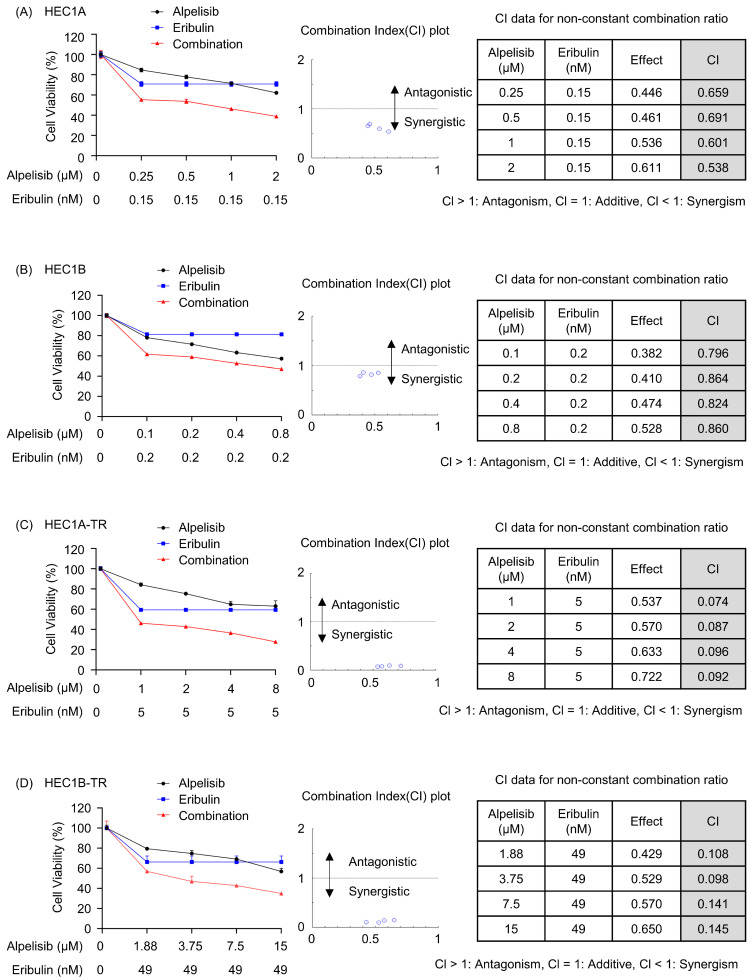
Alpelisib, a PI3K inhibitor, in combination with eribulin synergistically inhibits proliferation of both paclitaxel-sensitive and resistant endometrial cancer cells. (**A**–**D**) Paclitaxel-sensitive cells (HEC1A, HEC1B) and paclitaxel-resistant cells (HEC1A-TR, HEC1B-TR) were treated with alpelisib and eribulin at a non-constant ratio for 72 h. Then, cell proliferation was analyzed with MTT assay. The CI values were calculated using the Chou–Talalay method. CI < 1, CI > 1, and CI = 1 indicates synergism, antagonism, and additive effect, respectively. Three independently repeated experiments were performed with similar results.

The antiproliferative efficacy of all drugs, including paclitaxel, eribulin, and alpelisib, is summarized in Supplementary Table S2. The efficacy of eribulin and alpelisib as a single drug was also decreased in paclitaxel-resistant cells compared with that in parental cells. Based on our results that paclitaxel resistance was correlated with the activation of the PI3K/AKT pathway, we compared the anticancer efficacy of eribulin, alpelisib, and their combination at a fixed dose of eribulin and various concentrations of alpelisib (non-constant ratio) or various doses of eribulin and alpelisib (constant ratio) in parental and paclitaxel-resistant cells. The combination of the non-constant and constant ratios of eribulin and alpelisib showed synergistic antiproliferative effects on both parental and paclitaxel-resistant cells (Figure 2A–D and Supplementary Figure S1A–F). Taken together, these data indicate that the combination of alpelisib and eribulin has synergistic antiproliferative effects.

**Figure 3:**
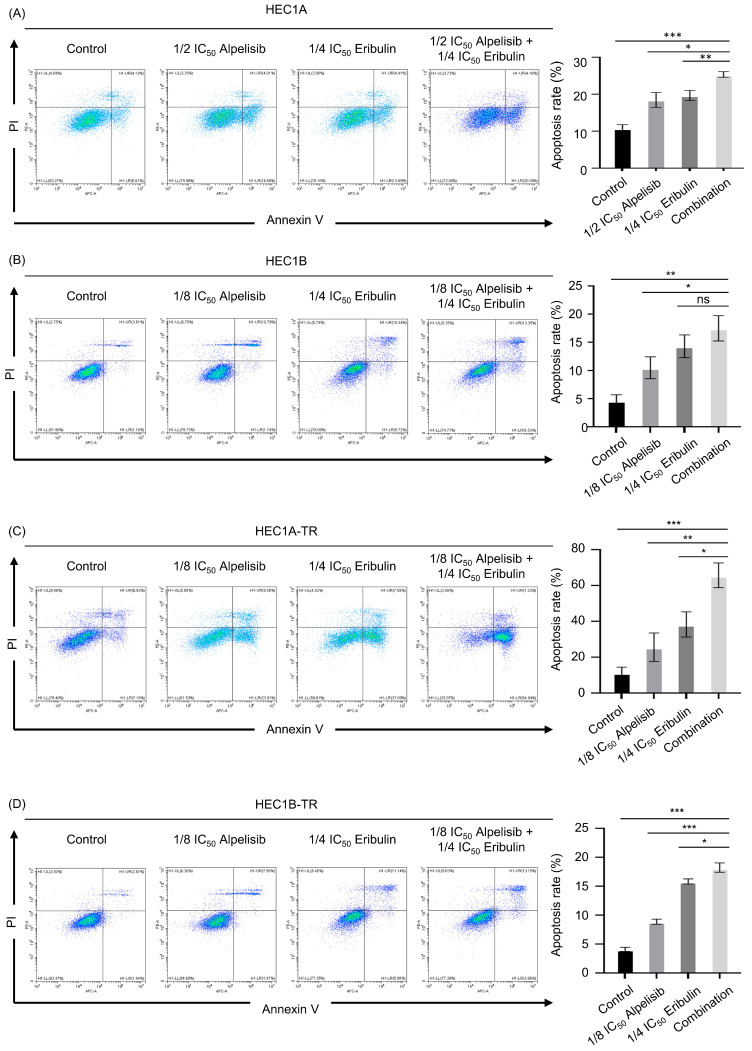
Synergistic effect of combined alpelisib and eribulin on apoptosis. Apoptosis assay with flow cytometry using annexin V-APC/PI staining in (**A**,**B**) paclitaxel-sensitive cells (HEC1A, HEC1B) and (**C**,**D**) paclitaxel-resistant cells (HEC1A-TR, HEC1B-TR) after treatment with alpelisib or eribulin and their combination for 72 h. The data shown are representative of three independent experiments. Data are presented as mean ± standard deviation from three independent experiments. *p*-values were calculated using Student’s *t*-test, indicating * *p* < 0.05, ** *p* < 0.01, and *** *p* < 0.001.

We further investigated whether the combination of two drugs affects the apoptosis of paclitaxel-resistant endometrial cancer cells using annexin V/PI staining. We tried to find out an appropriate concentration of each drug to demonstrate the optimal combination effect in each cell line. We adjusted drug concentrations as follows; the combination of even low doses of alpelisib (1/2 IC_50_ in parental cells; 1/8 IC_50_ in resistant cells) and eribulin (1/4 IC_50_ in both parental and resistant cells) induced more apoptosis compared with alpelisib or eribulin monotherapy in both parental and paclitaxel-resistant cells (Figure 3A–D and Supplementary Figure S2A).

### 3.3. The Combination of Alpelisib and Eribulin Suppresses the PI3K/AKT Pathway More Effectively Than Monotherapy in Both Paclitaxel-Sensitive and Paclitaxel-Resistant Endometrial Cancer Cells

**Figure 4:**
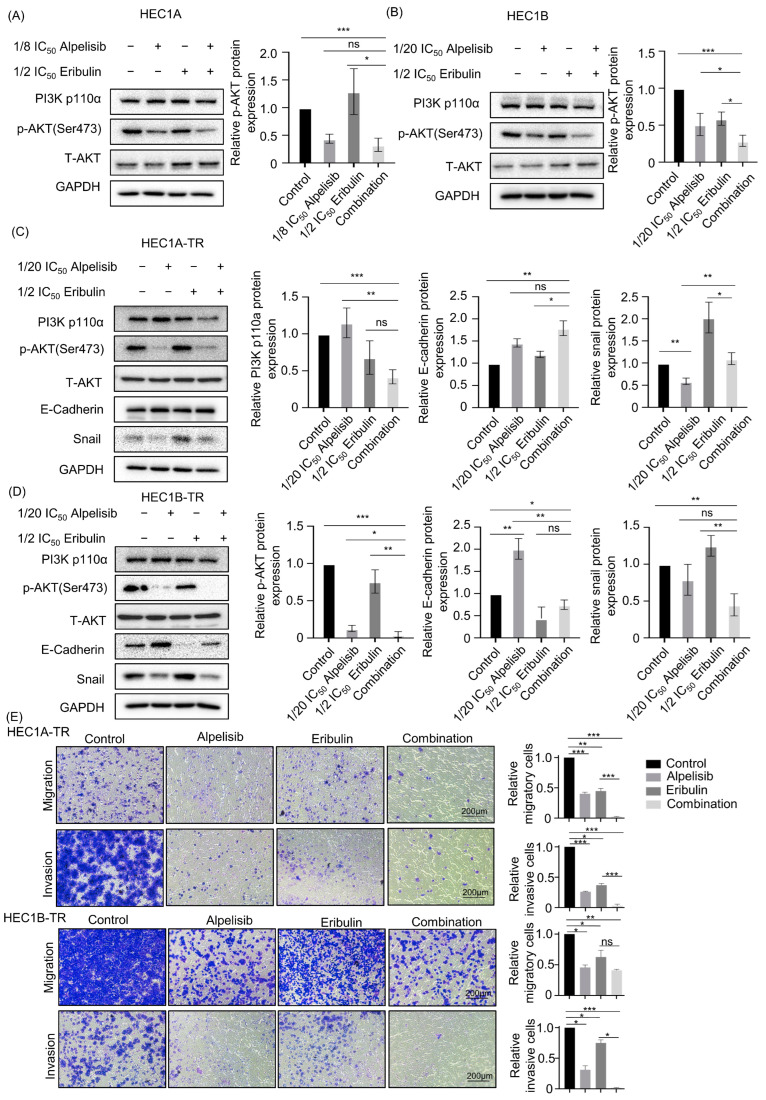
Combined alpelisib and eribulin suppresses PI3K/AKT pathway more effectively than monotherapy in paclitaxel-sensitive and -resistant endometrial cancer cells. (**A**,**B**) Western blots showing changes in the PI3K/AKT pathway-related genes after treatment with alpelisib or eribulin and their combination for 48 h in paclitaxel-sensitive cells (HEC1A, HEC1B). Data are presented as mean ± standard deviation from three independent experiments. *p*-values were calculated using Student’s *t*-test, indicating * *p* < 0.05 and *** *p* < 0.001. (**C**,**D**) Western blots showing changes in the PI3K/AKT pathway-related genes and EMT pathway-related genes in paclitaxel-resistant cells (HEC1A-TR, HEC1B-TR) after treatment with alpelisib, eribulin, or their combination for 48 h. Data are presented as mean ± standard deviation from three independent experiments. *p*-values were calculated using Student’s *t*-test, indicating * *p* < 0.05, ** *p* < 0.01, and *** *p* < 0.001. (**E**) Representative images of migration and invasion assay of HEC1A-TR and HEC1B-TR cells after treatment with IC_50_ concentration of alpelisib, eribulin, and their combination. The number of migratory and invading cells from three different non-overlapping 100 × microscopic fields is expressed as relative mean ± SD in the right panel. Independent sample *t*-test: * *p* < 0.05, ** *p* < 0.01, *** *p* < 0.001, Abbreviation: ns, not significant.

To investigate the mechanisms of synergism between alpelisib and eribulin in parental and paclitaxel-resistant endometrial cancer cells, we analyzed genes associated with the PI3K/AKT or EMT pathway using Western blot. Although, in parental cells (i.e., HEC1A, HEC1B, and AN3CA), alpelisib monotherapy decreased the expression of p-AKT, the combination of alpelisib and eribulin decreased the expression of p-AKT more than the monotherapy (Figure 4A,B and Supplementary Figure S2B). Consistent results were also observed in paclitaxel-resistant cells (i.e., HEC1A-TR and HEC1B-TR) (Figure 4C,D). We next examined whether monotherapy or the combination of alpelisib and eribulin could suppress EMT markers in paclitaxel-resistant cells. Alpelisib monotherapy decreased the expression of Snail and increased the expression of E-cadherin in paclitaxel-resistant cells. Furthermore, the combination of alpelisib and eribulin increased E-cadherin more and decreased the expression of Snail more than monotherapy (Figure 4C,D), indicating that the combination of alpelisib and eribulin reversed EMT in paclitaxel-resistant cells more effectively. Moreover, migration and invasion assays were performed to evaluate the impact of alpelisib, eribulin, and their combination. We found that the combination of alpelisib and eribulin significantly reduced the migratory and invasive capacity of paclitaxel-resistant as well as parental PIK3CA-mutated endometrial cancer cells (Figure 4E and Supplementary Figure S3A). In addition, to exclude the impact related to drug-induced proliferation or apoptosis in migration and invasion assays, an MTT assay was performed with the same concentration and treatment time of drugs, and each drug showed a minimum effect of drug-induced proliferation or apoptosis (Supplementary Figure S3B). Based on these data, we suggest that the combination of alpelisib and eribulin suppresses PIK3CA-mutated, paclitaxel-resistant endometrial cancer cells more effectively by inhibiting both the PI3K/AKT and EMT pathways.

### 3.4. The Combination of Alpelisib and Eribulin Regresses Paclitaxel-Resistant Endometrial Cancer Synergistically in a Xenograft Model

**Figure 5:**
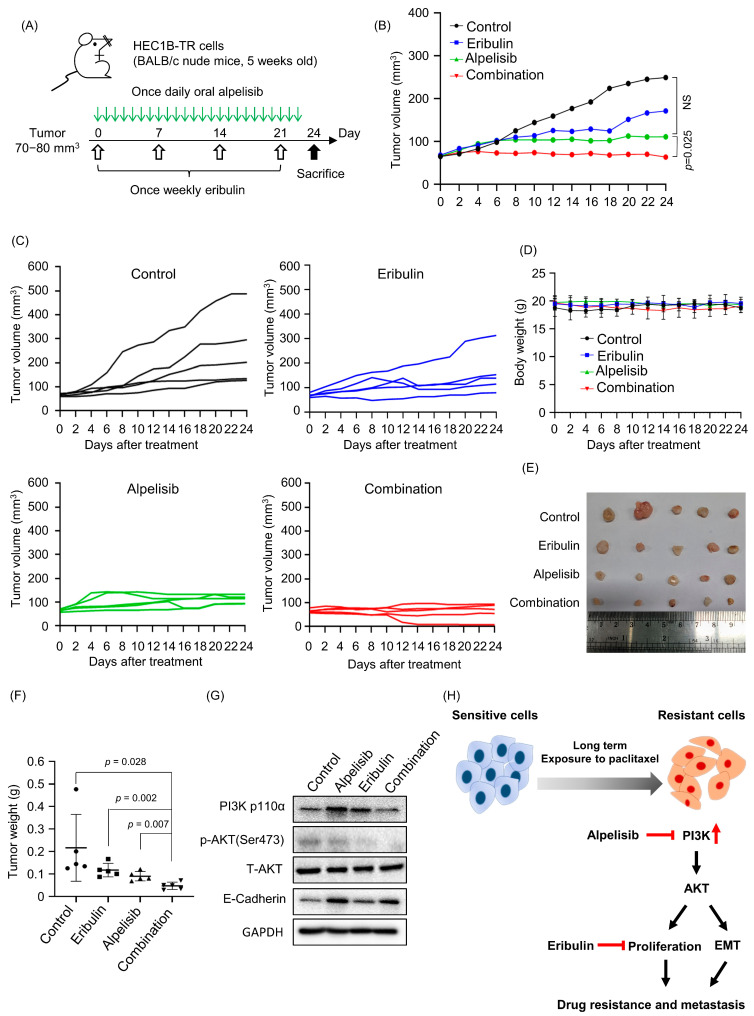
The combination of alpelisib and eribulin regresses paclitaxel-resistant endometrial cancer synergistically in a xenograft model. (**A**) Experimental scheme for in vivo study using acquired paclitaxel-resistant xenograft model. (**B**,**C**) Xenografted mice with HEC1B-TR cells were treated with alpelisib, eribulin, or a combination of both. (**B**) The average tumor growth inhibition curves of all mice in each group were drawn first. Then, (**C**) the tumor growth inhibition curves of individual mice were drawn in each group. *p*-values were calculated using Student’s *t*-test. (**D**) Average body weight of each treatment group mouse. Error bars represent the SD of 5 mice per group. (**E**) The xenografted tumors were excised 24 days following the start of the drug treatment, and each tumor’s images are displayed. (**F**) The tumor weights were parallel to the calculated tumor volumes. Data are presented as the mean ± SD. *p*-values were calculated using Student’s *t*-test. (**G**) Western blot using HEC1B-TR xenograft after 24 days showed greater inhibition of PI3K and p-AKT in combination treatment group. (**H**) Schematic diagram showing paclitaxel resistance mechanisms in PIK3CA-mutated endometrial cancer cells and overcoming strategy.

Based on our in vitro findings, we next sought to determine whether alpelisib, eribulin, or the combination of both drugs could suppress tumor growth. First, the paclitaxel-resistant endometrial cancer xenograft model was established using HEC1B-TR cells. As shown in Figure 5A, when a tumor reached 70–80 mm^3^, alpelisib was administrated every day (25 mg/kg) via oral gavage, whereas eribulin (1 mg/kg) was administrated once/week via intraperitoneal injection. The same dosing pattern was applied for the combination treatment. Pronounced tumor response was noted when alpelisib was combined with the eribulin drug, as opposed to alpelisib treatment alone (*p* = 0.025) (Figure 5B,C). Additionally, none of the treatments caused weight loss, suggesting the absence of systemic toxicity (Figure 5D). After 24 days of drug treatment, the mice were sacrificed, tumor tissues were collected (Figure 5E), and their weights were measured (Figure 5F). The weights of tumor tissues were consistent with those derived from measured tumor volumes. Finally, western blots using the tumor lysates showed remarkably decreased expression of PI3K and pAKT in the combination treatment compared with the monotherapy (Figure 5G), which is consistent with the in vitro results.

As illustrated schematically in Figure 5H, we found that activation of the PI3K/AKT pathway is associated with paclitaxel resistance and EMT progress. Combined treatment with alpelisib and eribulin overcame paclitaxel-resistant, PIK3CA-mutated endometrial cancer cells and reversed the EMT progress.

## 4. Discussion

The eventual escalation of endometrial carcinoma, in spite of being treated with paclitaxel/platinum as a first-line therapy in advanced or metastatic settings, emphasizes the significance of uncovering the mechanisms responsible for acquired resistance and developing next-line therapies. To explore the mechanisms of paclitaxel resistance in endometrial cancer, we developed robust preclinical models showing paclitaxel resistance, as validated by a >10-fold rise in the drug IC_50_. Our preclinical model confirmed that activation of the PI3K/AKT and EMT pathways is related to acquired paclitaxel resistance, in accordance with earlier investigations [9,15]. For the first time, we demonstrated that the inhibition of both PI3K/AKT activation and EMT progression with the combination of alpelisib and eribulin overcame paclitaxel resistance in endometrial cancer using our preclinical model. Considering the wide use of paclitaxel in advanced or metastatic endometrial cancer, our resistant model will be very valuable for developing drugs to overcome resistance.

First, we selected PIK3CA-mutated endometrial cancer cell lines as a parental model to generate acquired paclitaxel resistance. We observed that the PI3K pathway was triggered in the paclitaxel-resistant model compared with that in the paclitaxel-sensitive model, which parallels previous reports of associations between activation of the PI3K pathway and chemotherapy resistance, including paclitaxel resistance [16,17]. Considering that eribulin may not have cross resistance with paclitaxel, as in breast cancer [18], we selected eribulin as a combination partner with a PI3K inhibitor in paclitaxel-resistant, PI3K pathway-activated endometrial cancer. The combination of alpelisib and eribulin synergistically suppressed the proliferation of paclitaxel-resistant, PIK3CA-mutated endometrial cancer cells. Mechanistically, alpelisib inhibited the PI3K pathway, as demonstrated by a reduction in the expression of the total form of PI3K or p-AKT, potentially leading to genomic instability, as reported in previous studies [19]. Adding eribulin, a mitotic inhibitor, to this situation may enhance cell death. The synergistic mechanism of alpelisib and eribulin includes the simultaneous inhibition of the PI3K pathway and mitosis. To the best of our knowledge, this is the first study to combine a PI3K inhibitor and eribulin to overcome paclitaxel-resistant, PIK3CA-mutated endometrial cancer.

EMT progression is one of the mechanisms of drug resistance [20] and could be regulated by several cellular signaling pathways, including the PI3K/AKT pathway [21]. In our study, we demonstrated that EMT activation and migratory and invasive capacity were induced in paclitaxel-resistant endometrial cancer cells compared with that in their parental cells. Moreover, our combination therapy reversed EMT and migratory and invasive capacity in paclitaxel-resistant, PIK3CA-mutated endometrial cancer cells, particularly via the effect of alpelisib. Previously, other researchers have also reported that the combination of microtubule-targeting agents (e.g., paclitaxel and eribulin) and various PI3K/AKT/mTOR inhibitors suppressed the EMT pathway in ovarian cancer [10]. How the PI3K/AKT and EMT pathways in endometrial cancer cells are connected should be further studied.

As mentioned above, except for pembrolizumab with or without lenvatinib, subsequent lines of therapies after paclitaxel/platinum are limited. For platinum-sensitive patients who have relapsed with a treatment-free interval of ≥6 months following paclitaxel/platinum, retreatment with paclitaxel/platinum may be effective [22]. Endocrine therapy is widely used as a secondary option in unfit patients or low-grade, slow-progressing tumors [23]. Single-agent chemotherapy with doxorubicin may be used as a later-line therapy with limited evidence [24]. Bevacizumab may also be an option as a single agent and can be combined with chemotherapy [25]. Clinical evidence of these second- or later-line therapies in endometrial cancer is very limited, except for pembrolizumab, with or without lenvatinib. Regarding targeted therapy, for patients with human epidermal growth factor receptor 2 (HER2)-positive serous carcinomas, trastuzumab in combination with paclitaxel/carboplatin enhanced progression-free survival as a first- to fourth-line therapy in a phase II trial [26]. For patients with PI3K/AKT pathway activation, the mTOR inhibitors everolimus [27] or temsirolimus [28,29], the PI3K/mTOR dual inhibitor LY3023414 [30], and the AKT inhibitor MK2206 [31] only showed modest activity as a monotherapy or combination therapy. Of these, everolimus in combination with the aromatase inhibitor letrozole has shown a clinical benefit rate of 40% in a phase II trial [27] (Supplementary Table S3)**.** In this study, we first tested the effectiveness of the combination of a PI3K inhibitor and eribulin in a paclitaxel-resistant endometrial cancer preclinical model. As in this study, PI3K/AKT pathway-targeting agents should be further studied in combination with various anticancer drugs in endometrial cancer.

Further, the molecular classification of endometrial cancer, that is, polymerase epsilon (POLE) ultramutated, MSI hypermutated, copy-number (CN) low, and CN high, not only identifies prognosis but may also guide therapy. For instance, because the POLE ultamutated group shows an excellent prognosis, though the tumor is in high grade, stage I-II POLE-mutated patients do not need adjuvant therapy [2,32]. In addition, as previously mentioned, MSI-H patients with advanced or metastatic endometrial cancer benefit from immune checkpoint inhibitors. As we suggest in this paper, a PIK3CA or PTEN status may provide additional therapeutic implications in advanced or metastatic endometrial cancer, considering the relatively high frequency of PIK3CA mutation or PTEN loss, regardless of four molecular groups [33].

From the perspective of the toxicity of the combination of alpelisib and eribulin, the frequent toxicities of each drug include hyperglycemia (59%) and diarrhea (56%) for alpelisib [34] and neutropenia (52%) and peripheral neuropathy (35%) for eribulin [35]. Frequent toxicities of both drugs are not overlapped like this. Furthermore, in an animal study, the combination of alpelisib and eribulin did not cause weight loss, demonstrating the absence of systemic toxicity. Taken together, the combination of alpelisib and eribulin may have merits in terms of toxicity.

## 5. Conclusions

In conclusion, this study demonstrated that the combination of alpelisib and eribulin enhanced antitumor activity in a paclitaxel-resistant, PIK3CA-mutated endometrial cancer preclinical model. This combination warrants further investigation in a clinical trial, with a particular aim to overcome chemotherapy resistance in endometrial cancer.

## Data Availability

All the data of this study are available upon request.

## Supplementary Materials

The following supporting information can be downloaded at: https://www.mdpi.com/article/10.3390/cancers15194887/s1, Figure S1: Cell proliferation MTT assay of paclitaxel-sensitive (HEC1A, HEC1B, AN3CA) and paclitaxel-resistant cells (HEC1A-TR, HEC1B-TR) after treatment with alpelisib and eribulin at constant and non-constant combination ratios, Figure S2: Apoptosis assay (annexin V-APC/PI) and western blot analysis of PI3K/AKT in paclitaxel-sensitive cells (AN3CA) after treatment with alpelisib or eribulin and their combination, Figure S3: Migration and invasion assay of HEC1A and HEC1B cells; MTT assay of HEC1A, HEC1A-TR, HEC1B and HEC1B-TR cells; Figure S4: Uncropped western blots; Table S1: List of primary and secondary antibodies used in western blot, Table S2: Summary of IC_50_ of endometrial cancer cell lines for several drugs, Table S3: Summary of clinical trials with PI3K-targeting agents in metastatic endometrial cancers [36,37].
