# Supplementary material for: Combined PI3K Inhibitor and Eribulin Enhances Anti-Tumor Activity in Preclinical Models of Paclitaxel-Resistant, PIK3CA-Mutated Endometrial Cancer
Source: Cancers (Basel). 2023 Oct 8;15(19):4887. doi: 10.3390/cancers15194887 (PMC10571568; doi:10.3390/cancers15194887)
Supplement: Supplementary file 1 [file cancers-15-04887-s001.zip › cancers-2605248-supplementary.pdf]

Supplementary Materials: Combined PI3K Inhibitor and Eribulin Enhances Anti-Tumor Activity in Preclinical Models of Paclitaxel-Resistant, PIK3CA-Mutated Endometrial Cancer

Yeong Gyu Jeong, Nar Bahadur Katuwal, Min Sil Kang, Mithun Ghosh, Sa Deok Hong, Seong Min Park, Seul-Gi Kim, Tae Hoen Kim, Yong Wha Moon

(A)

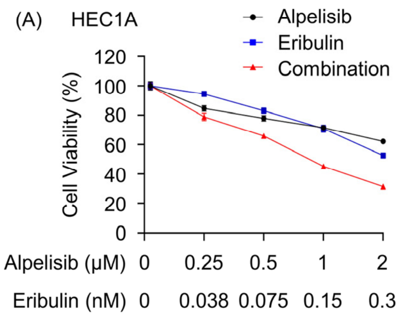

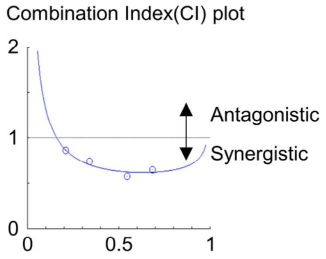

CI data for constant combination ratio

Alpelisib (μM)	Eribulin (nM)	Effect	CI
0.25	0.038	0.211	0.863
0.5	0.075	0.341	0.739
1	0.15	0.548	0.573
2	0.3	0.685	0.645

CI > 1: Antagonism, CI = 1: Additive, CI < 1: Synergism

(B)

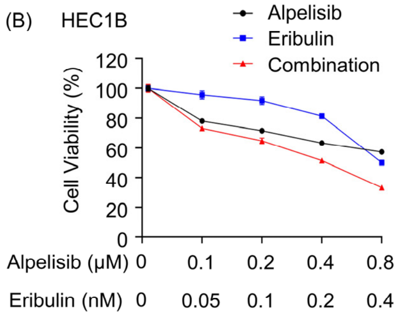

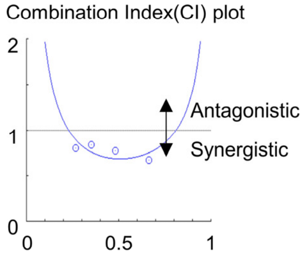

CI data for constant combination ratio

Alpelisib (μM)	Eribulin (nM)	Effect	CI
0.1	0.05	0.267	0.807
0.2	0.1	0.352	0.846
0.4	0.2	0.485	0.780
0.8	0.4	0.667	0.679

CI > 1: Antagonism, CI = 1: Additive, CI < 1: Synergism

(C)

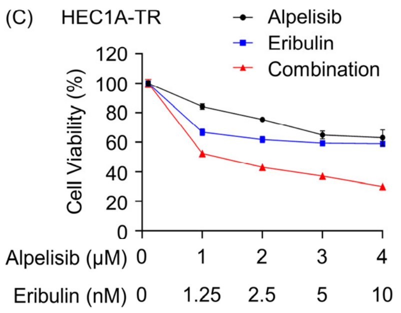

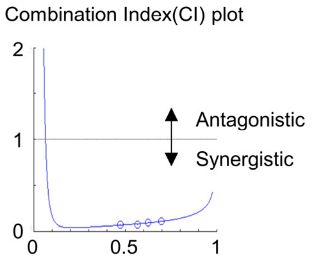

CI data for constant combination ratio

Alpelisib (μM)	Eribulin (nM)	Effect	CI
1	1.25	0.474	0.074
2	2.5	0.567	0.077
4	5	0.627	0.099
8	10	0.699	0.112

CI > 1: Antagonism, CI = 1: Additive, CI < 1: Synergism

(D)

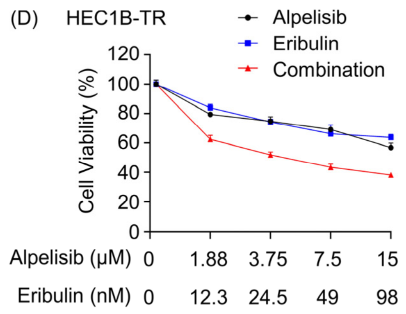

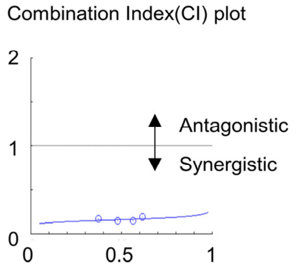

CI data for constant combination ratio

Alpelisib (μM)	Eribulin (nM)	Effect	CI
1.88	12.3	0.374	0.170
3.75	24.5	0.479	0.145
7.5	49	0.564	0.148
15	98	0.618	0.190

CI > 1: Antagonism, CI = 1: Additive, CI < 1: Synergism

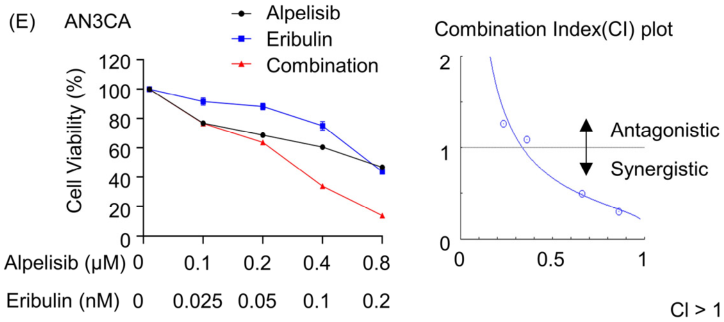

CI data for constant combination ratio

Alpelisib (μM)	Eribulin (nM)	Effect	CI
0.1	0.025	0.234	1.262
0.2	0.05	0.363	1.089
0.4	0.1	0.662	0.493
0.8	0.2	0.861	0.304

CI > 1: Antagonism, CI = 1: Additive, CI < 1: Synergism

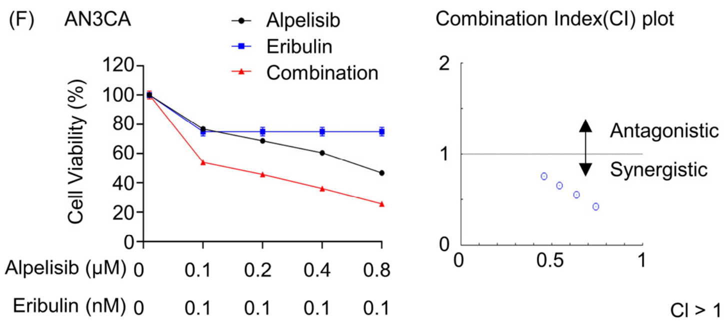

CI data for non-constant combination ratio

Alpelisib (μM)	Eribulin (nM)	Effect	CI
0.1	0.1	0.460	0.758
0.2	0.1	0.543	0.657
0.4	0.1	0.639	0.551
0.8	0.1	0.744	0.427

CI > 1: Antagonism, CI = 1: Additive, CI < 1: Synergism

Figure S1. Cell proliferation MTT assay of paclitaxel-sensitive (HEC1A, HEC1B, AN3CA) and paclitaxel-resistant cells (HEC1A-TR, HEC1B-TR) after treatment with alpelisib and eribulin at constant and non-constant combination ratios (A-D) Cell proliferation assay (MTT) of paclitaxel-sensitive cells (HEC1A, HEC1B) and paclitaxel-resistant cells (HEC1A-TR, HEC1B-TR) after treatment with alpelisib and eribulin at a constant combination ratio. (E-F) Cell proliferation assay (MTT) of paclitaxel-sensitive cells (AN3CA) after treatment with alpelisib and eribulin (E) at a constant combination ratio or (F) at a non-constant ratio for 72h. The CI values were calculated by the Chou-Talalay method. CI < 1, CI > 1, and CI = 1 indicate synergism, antagonism, and additive effect, respectively. Three independently repeated experiments were performed with similar results.

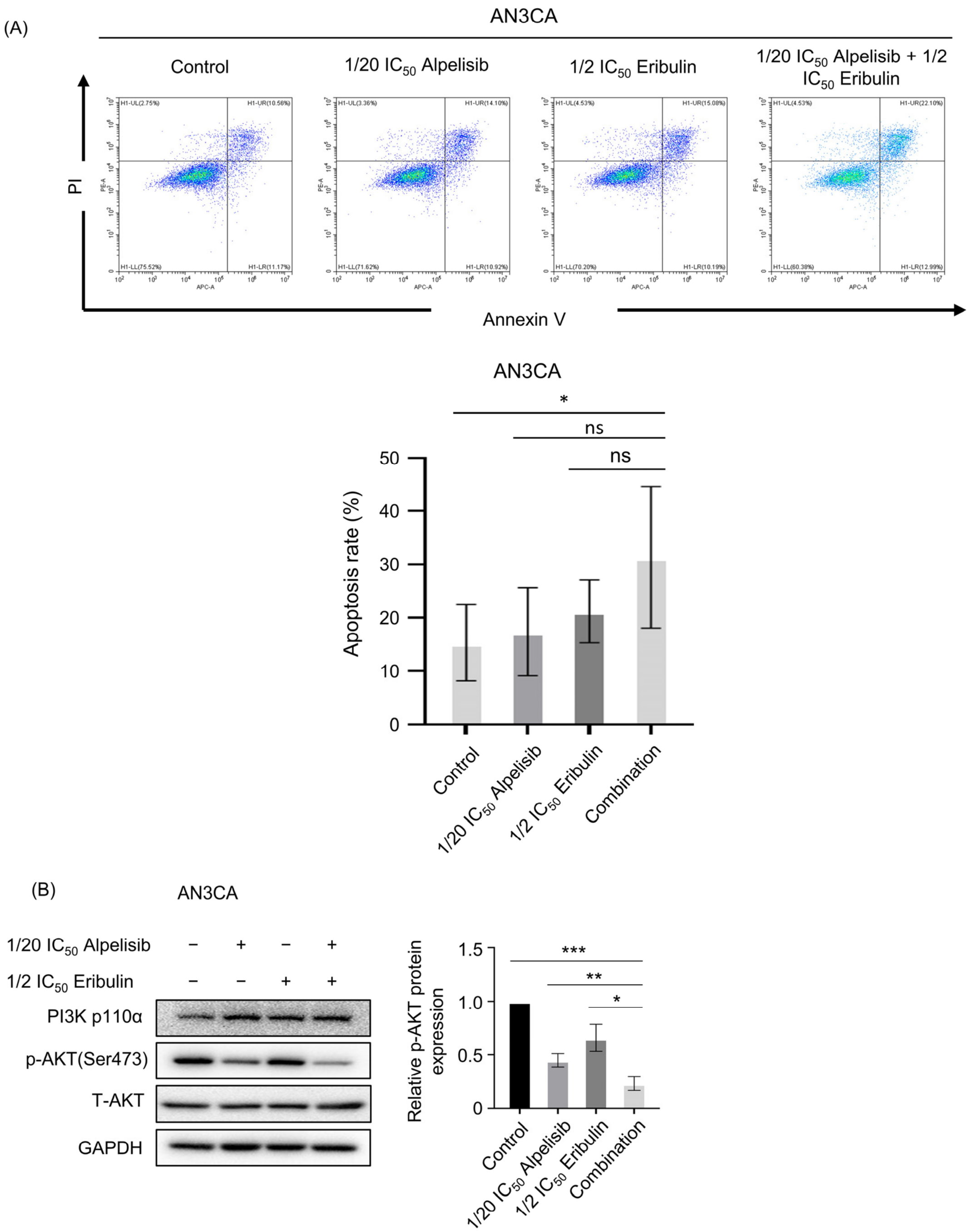

Figure S2. Apoptosis assay (annexin V-APC/PI) and western blot analysis of PI3K/AKT in paclitaxel-sensitive cells (AN3CA) after treatment with alpelisib or eribulin and their combination (A) Apoptosis assay with flow cytometry using annexin V-APC/PI staining in paclitaxel-sensitive cells (AN3CA) after treatment with alpelisib or eribulin and their combination for 72h. The data shown are representative of three independent experiments. Data are presented as mean ± standard deviation from three independent experiments. *P*-values were calculated by Student's t-test, indicating * $p < 0.05$, ** $p < 0.01$, and *** $p < 0.001$. (B) Western blots showing changes in the PI3K/AKT pathway-related genes in paclitaxel-sensitive cells (AN3CA) after treatment with alpelisib or eribulin and their combination for 48h. Data are presented as mean ± standard deviation from three independent experiments. *P*-values were calculated by Student's t-test, indicating * $p < 0.05$, ** $p < 0.01$, and *** $p < 0.001$.

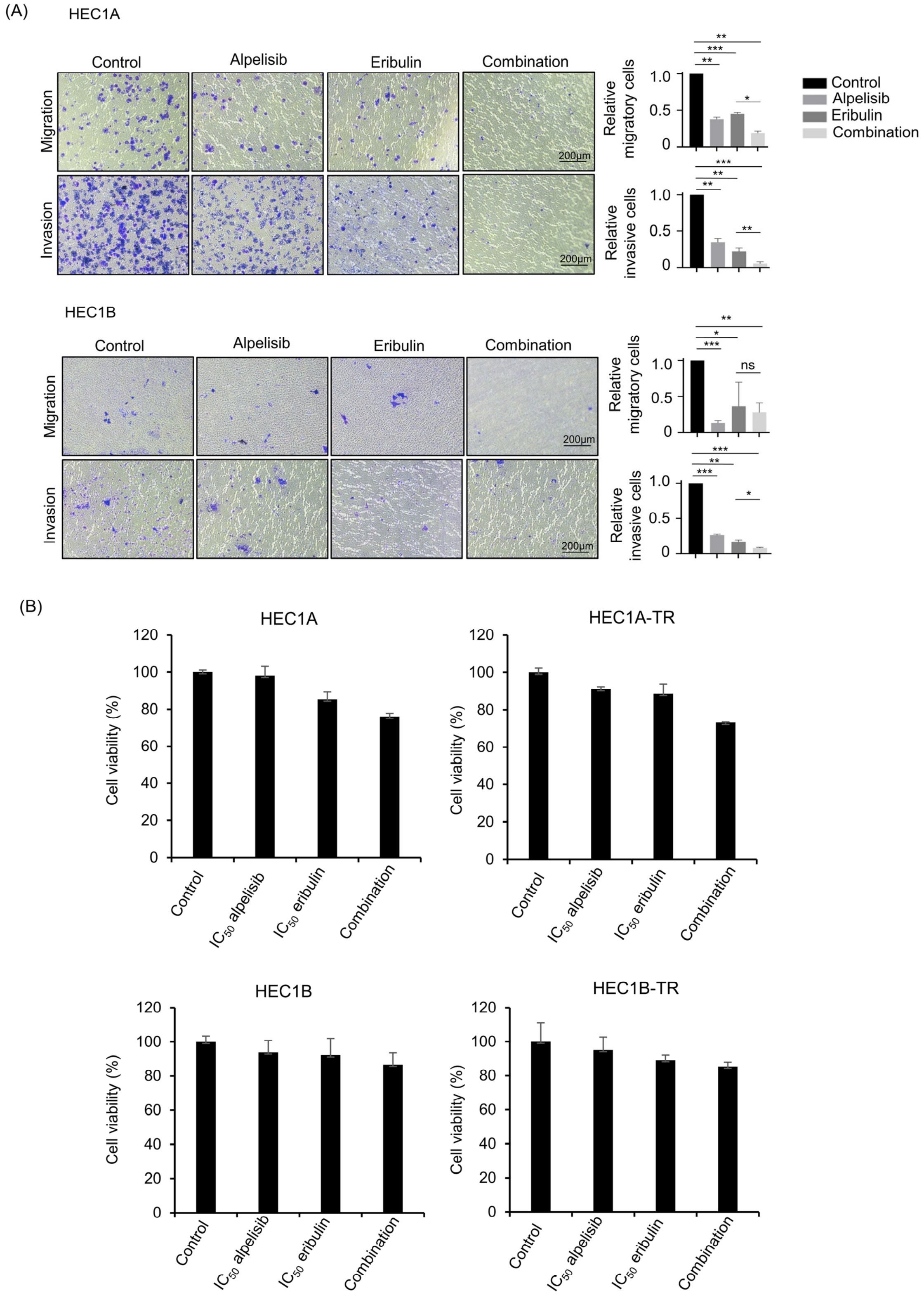

Figure S3. Migration and invasion assay of HEC1A and HEC1B cells; MTT assay of HEC1A, HEC1A-TR, HEC1B and HEC1B-TR cells (A) Representative images of migration and invasion assay of HEC1A and HEC1B cells after treatment with IC_{50} concentration of alpelisib, eribulin and their combination. The number of migratory and invading cells from three different non-overlapping 100 × microscopic fields is expressed as relative mean ± SD in the right panel. Independent sample t-test: *$p < 0.05$, **$p < 0.01$, ***$p < 0.001$, Abbreviation: ns, not significant. (B) MTT assay of HEC1A, HEC1A-TR, HEC1B and HEC1B-TR cells with their respective IC_{50} concentration of alpelisib, eribulin and combination for 24 h.

Figure 4.

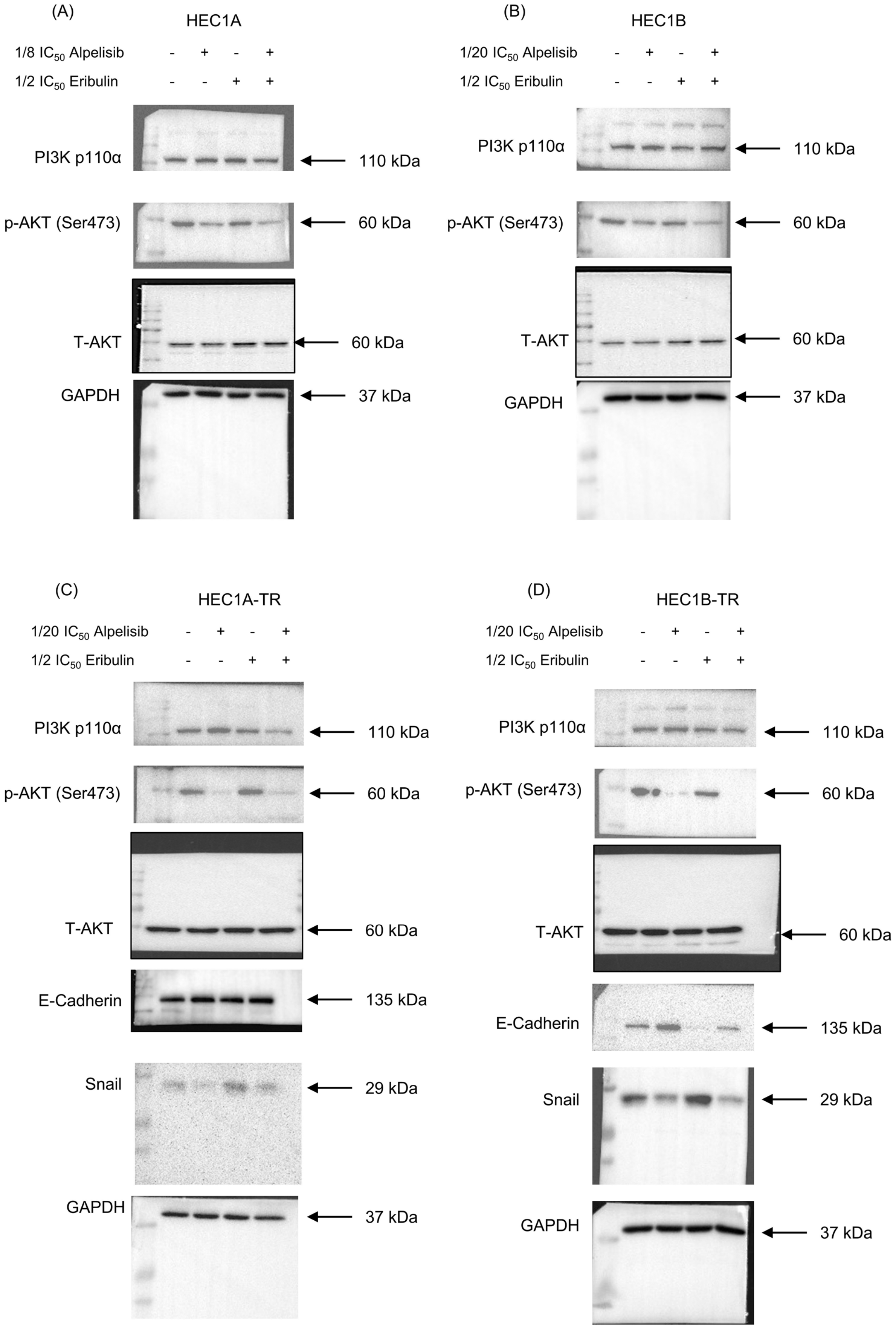
(A)
HEC1A
1/8 IC_{50} Alpelisib
- + - +
1/2 IC_{50} Eribulin
- - + +
PI3K p110α
110 kDa
p-AKT (Ser473)
60 kDa
T-AKT
60 kDa
GAPDH
37 kDa
(B)
HEC1B
1/20 IC_{50} Alpelisib
- + - +
1/2 IC_{50} Eribulin
- - + +
PI3K p110α
110 kDa
p-AKT (Ser473)
60 kDa
T-AKT
60 kDa
GAPDH
37 kDa
(C)
HEC1A-TR
1/20 IC_{50} Alpelisib
- + - +
1/2 IC_{50} Eribulin
- - + +
PI3K p110α
110 kDa
p-AKT (Ser473)
60 kDa
T-AKT
60 kDa
E-Cadherin
135 kDa
Snail
29 kDa
GAPDH
37 kDa
(D)
HEC1B-TR
1/20 IC_{50} Alpelisib
- + - +
1/2 IC_{50} Eribulin
- - + +
PI3K p110α
110 kDa
p-AKT (Ser473)
60 kDa
T-AKT
60 kDa
E-Cadherin
135 kDa
Snail
29 kDa
GAPDH
37 kDa

Figure 5.

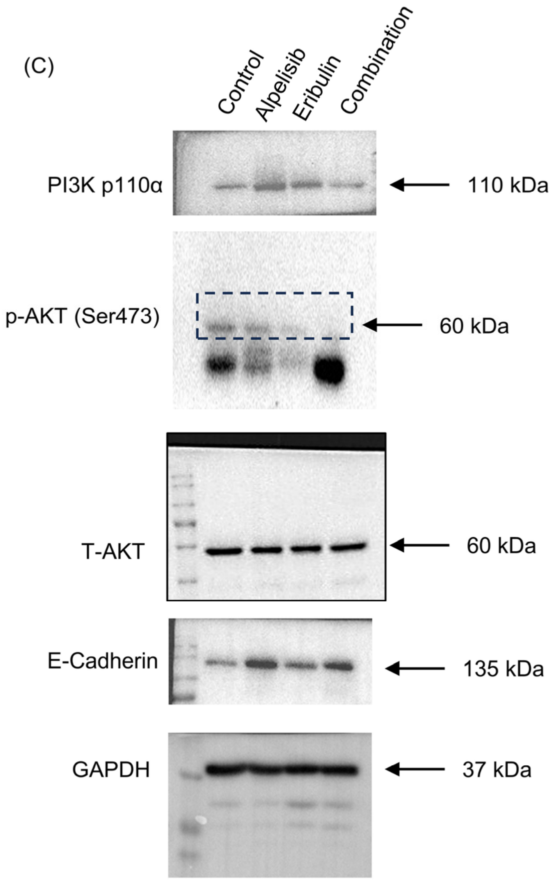

Figure S2.

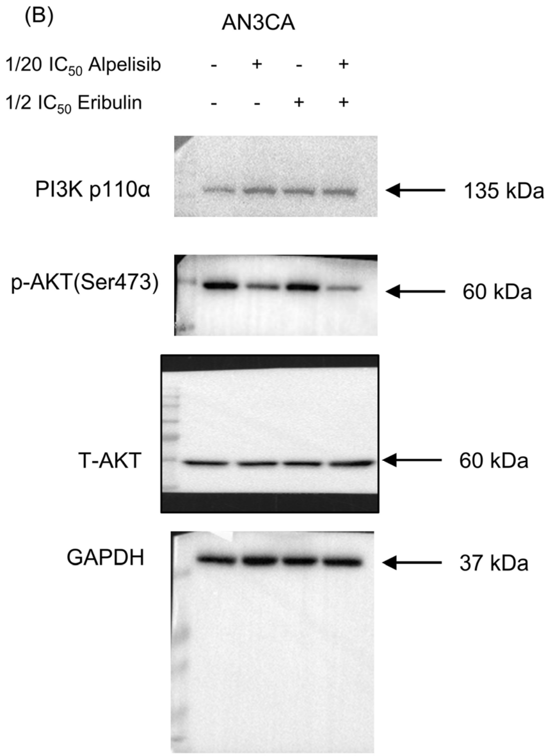

Figure S4. Uncropped western blots.

Table S1. List of primary and secondary antibodies used in western blot.

Antibody	Host species	Dilution	Company (catalog no.)
PI3K p110a	Rabbit	1:1000	4249S, Cell Signaling, Danvers, Massachusetts, USA
p-AKT (Ser473)	Rabbit	1:1000	4060S, Cell Signaling, Danvers, Massachusetts, USA
AKT	Rabbit	1:1000	9272S, Cell Signaling, Danvers, Massachusetts, USA
PTEN	Rabbit	1:1000	9559S, Cell Signaling, Danvers, Massachusetts, USA
E-Cadherin	Rabbit	1:1000	3195S, Cell Signaling, Danvers, Massachusetts, USA
Snail	Rabbit	1:1000	3879S, Cell Signaling, Danvers, Massachusetts, USA
ZEB1	Rabbit	1:1000	ab124512, abcam
LAMC2	Rabbit	1:1000	sc-28330, Santa Cruz, Dallas, Texas, USA
GAPDH	Rabbit	1:1000	2118S, Cell Signaling, Danvers, Massachusetts, USA
Anti-Rabbit HRP	Goat	1:5000	GTX213110-01, GeneTex, CA 92606, USA

Table S2. Summary of IC_{50} of endometrial cancer cell lines for several drugs.

	Paclitaxel IC_{50} (nM)	Incubation time (h)	Alpelisib IC_{50} (uM)	Incubation time (h)	Eribulin IC_{50} (nM)	Incubation time (h)
HEC1A	6.8	72	2	72	0.3	72
HEC1A-TR	111	72	8	72	10	72
HEC1B	15	72	0.8	72	0.4	72
HEC1B-TR	160	72	15	72	98	72
AN3CA	3.6	72	0.8	72	0.2	72

Table S3. (A) Summary of clinical trials with PI3K-targeting agents in metastatic endometrial cancers.

Author (reference)	Year	Phase	N	Setting	PI3K-targeting agent (target)/ Combinators	Median PFS (month)	Median OS (month)
Oza [1]	2011	II	60	1st line: 27<br>2nd line or more: 33	Temsirolimus (mTOR)	7.33 mo in 1st line<br>3.25 mo in 2nd or more line	OS not-mentioned
Alvarez [2]	2013	II	49	2nd ~3rd line	Temsirolimus (mTOR)/bevacizumab	5.6 mo	16.9 mo
Oza [3]	2015	II	64	2nd ~3rd line	Arm 1: Ridaforolimus (mTOR)	3.6 mo	10.0 mo
			66		Arm2: Progestin or any chemotherapy	1.9 mo; P=0.008	9.6 mo; P=0.604
Slomovitz [4]	2015	II	38	1st line: 3<br>2nd ~3rd line: 35	Everolimus (mTOR)/letrozole	3.0 mo	14 mo
Aghajanian [5]	2018	II	115	1st line	Temsirolimus (mTOR)/<br>paclitaxel/carboplatin	PFS, not-mentioned<br>HR 1.22 (92% CI: 0.96-1.55) in temsirolimus arm, compared with historical control	OS, not-mentioned<br>HR 0.99 (92% CI: 0.78-1.26) in temsirolimus arm, compared with historical control
Myers [6]	2020	II	36	2nd ~3rd line	MK2206 (AKT)	1.7 mo in PIK3CA mutated pts, 2.5 mo in PIK3CA wild type pts	8.4 mo in PIK3CA mutated pts, 11.1 mo in PIK3CA wild type pts
Rubinstein [7]	2020	II	28	2nd~4th line	LY3023414 (dual PI3K/mTOR)	2.5 mo	9.2 mo

Abbreviations: N, number of patients; PFS, progression-free survival; OS, overall survival; HR, hazard ratio; mo, month; CI, confidence interval; pts, patients

References

1. Oza, A.M.; Elit, L.; Tsao, M.S.; Kamel-Reid, S.; Biagi, J.; Provencher, D.M.; Gotlieb, W.H.; Hoskins, P.J.; Ghatage, P.; Tonkin, K.S.; et al. Phase II study of temsirolimus in women with recurrent or metastatic endometrial cancer: a trial of the NCIC Clinical Trials Group. *J Clin Oncol* **2011**, *29*, 3278-3285.
2. Alvarez, E.A.; Brady, W.E.; Walker, J.L.; Rotmensch, J.; Zhou, X.C.; Kendrick, J.E.; Yamada, S.D.; Schilder, J.M.; Cohn, D.E.; Harrison, C.R.; et al. Phase II trial of combination bevacizumab and temsirolimus in the treatment of recurrent or persistent endometrial carcinoma: A Gynecologic Oncology Group study. *Gynecol. Oncol.* **2013**, *129*, 22–27, https://doi.org/10.1016/j.ygyno.2012.12.022.
3. Oza, A.M.; Pignata, S.; Poveda, A.; McCormack, M.; Clamp, A.; Schwartz, B.; Cheng, J.; Li, X.; Campbell, K.; Dodion, P.; et al. Randomized Phase II Trial of Ridaforolimus in Advanced Endometrial Carcinoma. J Clin Oncol 2015, 33, 3576-3582.
4. Slomovitz, B.M.; Jiang, Y.; Yates, M.S.; Soliman, P.T.; Johnston, T.; Nowakowski, M.; Levenback, C.; Zhang, Q.; Ring, K.; Munsell, M.F.; et al. Phase II study of everolimus and letrozole in patients with recurrent endometrial carcinoma. *J Clin Oncol* **2015**, *33*, 930-936.
5. Aghajanian, C.; Filiaci, V.; Dizon, D.S.; Carlson, J.W.; Powell, M.A.; Secord, A.A.; Tewari, K.S.; Bender, D.P.; O'Malley, D.M.; Stuckey, A.; et al. A phase II study of frontline paclitaxel/carboplatin/bevacizumab, paclitax-el/carboplatin/temsirolimus, or ixabepilone/carboplatin/bevacizumab in advanced/recurrent endometrial cancer. Gynecol Oncol 2018, 150, 274-281.
6. Myers, A.P.; Konstantinopoulos, P.A.; Barry, W.T.; Luo, W.; Broaddus, R.R.; Makker, V.; Drapkin, R.; Liu, J.; Doyle, A.; Horowitz, N.S.; et al. Phase II, 2-stage, 2-arm, PIK3CA mutation stratified trial of MK-2206 in recurrent endometrial cancer. *Int J Cancer* **2020**, *147*, 413-422.
7. Rubinstein, M.M.; Hyman, D.M.; Caird, I.; Won, H.; Soldan, K.; Seier, K.; Iasonos, A.; Tew, W.P.; O'Cearbhaill, R.E.; Grisham, R.N.; et al. Phase 2 study of LY3023414 in patients with advanced endometrial cancer harboring activating mutations in the PI3K pathway. *Cancer* **2020**, *126*, 1274-1282.
